# Phenotypic Characterization of Circulating CD4^+^ T Cells in ANCA-Associated Vasculitis

**DOI:** 10.1155/2018/6984563

**Published:** 2018-10-30

**Authors:** Sandra Lilliebladh, Åsa Johansson, Åsa Pettersson, Sophie Ohlsson, Thomas Hellmark

**Affiliations:** ^1^Department of Clinical Sciences Lund, Nephrology, Lund University, Skåne University Hospital, Lund, Sweden; ^2^Department of Laboratory Medicine in Lund, Haematology and Transfusion Medicine, Lund University, Skåne University Hospital, Lund, Sweden

## Abstract

T cell-mediated immune responses are thought to play an important role in the pathogenesis of anti-neutrophil cytoplasmic antibody- (ANCA-) associated vasculitides (AAV). CD4^+^ T cells can be divided into subsets depending on their expression of chemokine receptors. In this study, different CD4^+^ T cell populations in patients with AAV were analysed and compared to healthy blood donors as well as therapy controls. 18 patients with active AAV, 46 in remission, 21 healthy controls (HBD), and 15 therapy controls (TC) were enrolled. CD4^+^ T cells were divided into Th1, Th2, and Th17 cells and further subdivided into naïve, central memory, effector memory, and effector cells. Regulatory T cells were also analysed. Concentrations of cytokines and chemokines produced by the respective CD4^+^ T cell subset in plasma from 33 of the patients were measured by ELISA and compared to HBD. Clinical data were collected on all patients. CCL20 concentrations and percentages of Th17 cells (*p* = 0.019) were elevated in AAV patients compared to HBD. AAV patients had lower percentages of naïve CD4^+^ T cells (*p* = 0.0016) and a corresponding increase in proportion of effector memory CD4^+^ T cells when comparing to HBD (*p* = 0.027). Therapy controls showed similar results as AAV patients. In this study, we found that CD4^+^ T cell phenotype distribution is altered in AAV patients, in line with previously published work. However, no differences were found between AAV patients and TC, stressing the importance of treatment impact on this kind of studies.

## 1. Introduction

The anti-neutrophil cytoplasmic autoantibody- (ANCA-) associated vasculitides (AAV) are a group of autoimmune diseases characterized by necrotizing inflammation predominantly in small blood vessels and comprise granulomatosis with polyangiitis (GPA), microscopic polyangiitis (MPA), and eosinophilic granulomatosis with polyangiitis (EGPA) [1, 2]. Especially GPA and MPA have a strong association with ANCA, GPA predominantly with ANCA targeting proteinase 3 (PR3-ANCA), and MPA with ANCA against myeloperoxidase (MPO-ANCA) [3]. AAV often presents clinically as a systemic disease. Although the inflammation can affect any organ in the body, the kidneys together with upper and lower airways are most frequently involved. Most of the current therapies are associated with severe side effects, and relapse rates are, despite treatment, generally high.

The pathogenesis of AAV is multifactorial, including genetic and environmental factors such as infections and drugs, but the exact mechanisms still remain elusive [4]. The pathogenicity of PR3-ANCA and MPO-ANCA is debated, but it is likely that these autoantibodies to some, perhaps varying, extent are pathogenic. Activation of the complement system, especially through the alternative pathway, is also thought to contribute to the vasculitis process [5, 6].

CD4^+^ T cells (Th) can be divided into different subsets based on their cytokine profiles, e.g., Th1, Th2, and Th17, but also Th9 cells, Th22 cells, and follicular helper T cells. For instance, Th1 cells are characterized by IFN-*γ* production and are presumed to have a proinflammatory role as well as a role in fighting infections. Th2 cells are of importance in allergic inflammations and parasite infections, e.g., by secreting IL-4 and IL-5. Th17 cells produce IL-17(A-F), IL-21, and IL-22. Th17 cells have been suggested to be implicated in several autoimmune diseases such as psoriasis, inflammatory bowel disease, and ankylosing spondylitis [7–10].

CD4^+^ T cells can also be divided into different subsets based on their ability to proliferate and/or effector function, i.e., naïve, stem cell memory, central memory (CM), transitional memory (TM), effector memory (EM), and terminal effector (Eff) Th cells. The naïve cells have the highest proliferation potential, lymphoid homing profile, self-renewal capacity, and multipotency and the terminal effector cells the lowest. Reversely, the terminal effector cells exhibit the highest peripheral homing profile, effector function, and antigen dependence.

CD4^+^ T cells are thought to play a substantial role in the development of granulomatous inflammation and tissue injury in AAV [11–13]. However, the role of various subtypes of CD4^+^ T cells in AAV has not yet been fully established. Earlier studies have suggested a Th1-dominated immune response in GPA [14, 15], while others have suggested a dominant Th2 cell-driven immune response [16]. There are a few reports indicating a role for Th17 in AAV, e.g., increased percentage of IL-17-producing CD4^+^ T cells in GPA patients after in vitro stimulation with the autoantigen PR3 [17]. In line with this finding, Nogueira et al. found increased levels of IL-17 and IL-23 during the acute phase of AAV and increased number of IL-17-producing autoantigen-specific cells during the convalescence phase. No differences in IFN-*γ*-producing cells were observed [18]. Results from animal models support an important role of IL-17 in vasculitis. IL-17-deficient mice developed less severe immune-mediated kidney injury [19] and MPO-vasculitis [20]. Moreover, higher percentages of circulating EM cells were found in GPA patients in remission compared to healthy blood donors (HBD) [21].

CD4^+^ T cells can also be differentiated into regulatory T cells (Tregs), which are important in maintenance of immunologic tolerance and limitation of inflammatory responses. Functionally defect Tregs have been reported in patients with GPA [22].

In this study, we investigate the frequency of various CD4^+^ T cell subsets as well as effector cytokines and chemokines in plasma from AAV patients, in remission or with active disease, in relation to healthy blood donors and therapy controls. The aim is to increase our understanding of the CD4^+^ T cell involvement in AAV.

## 2. Materials and Methods

### 2.1. Study Population

Blood samples were collected from 64 patients with AAV. Forty-six of these patients were in remission, and 18 had an active disease. 38 of the patients were diagnosed with GPA, 22 were diagnosed with MPA, and 4 were diagnosed with EGPA. 35 of the patients were PR3-ANCA-positive, 25 MPO-ANCA-positive, and 4 ANCA-negative. Blood samples were also collected from 21 healthy controls (HBD) and 15 patients with a kidney transplant due to a noninflammatory disease (therapy control, TC). See Table 1 for descriptive statistics of the patients with GPA and MPA and Table 2 for descriptive statistics of the whole AAV group, HBD, and TC. There was no matching between the different groups regarding age or gender. The small group of EGPA patients consisted of two women and two men; three of the patients were ANCA-negative, and one had MPO-ANCA. None of the patients with EGPA had active disease. The EGPA patients are included in the analyses of the AAV group and when dividing the patients according to ANCA specificity.

eGFR was calculated for AAV patients and TC using the MDRD equation (http://www.mdrd.com). The studies were conducted with permission from the Ethical Committee, Lund University, Sweden.

For the ELISA analysis, additional EDTA-plasma were obtained from the 33 patients first included and compared to EDTA-plasma from the 13 first included HBD. 26 of these patients were in remission, and 7 had an active disease. Twenty-one of the patients were diagnosed with GPA, 10 with MPA, and 2 with EGPA.

Diagnosis was based on criteria adapted from the 1994/2012 Chapel Hill disease definitions and the American College of Rheumatology's classification criteria [1, 2]. Patients were excluded if they had a malignancy, an ongoing infection, or a coexisting autoimmune condition. Clinical data were collected on all patients. Disease activity was estimated by using Birmingham Vasculitis Activity Score v3 (BVAS) [23]. Active disease was defined as having a BVAS score of more than 0. Relapse was defined as a BVAS score over 0 in combination with an increased immunosuppressive therapy. In order to score the overall damage due to vasculitis, Vasculitis Damage Index (VDI) was used [24]. Localized disease was defined as involvement of only one organ system, besides general symptoms and signs. Serum levels of PR3-ANCA and MPO-ANCA were measured by ELISA-based methods including capture technique at Wieslab©, Euro Diagnostica AB (Lund, Sweden). All patients, HBD, and TC gave informed consent.

HBD were subjects that volunteered to participate in the study as controls, with no known inflammatory or chronic disease. Median age was 56.0 years (IQR: 45.3, 60.2), and the group consisted of 11 women and 10 men. TC were 4 women and 11 men from our outpatient clinic, with no inflammatory diseases, that had received a kidney transplant. Median age was 49.4 years, and median eGFR was 55.8 mL/min/1.73 m^2^. 11 out of 15 therapy controls were treated with prednisolone (median 5 mg/day, range: 2.5, 5), 11 with tacrolimus (median: 4 mg/day, range: 0.5, 5), 2 with everolimus (median: 1.2 mg/day, range: 1.0, 2.2), 9 with mycophenolate mofetil (median: 1500 mg/day, range: 750, 2000), 4 with cyclosporine (median: 137.5 range: 50, 200), and 2 with azathioprine (median: 50 mg/day range: 50, 50), and one of the TC was treated with mycophenolic acid (720 mg/day) (Table 2).

### 2.2. Phenotypic Characterization of CD4^+^ T Cells: Flow Cytometry

Venous blood was obtained in heparin tubes (BD Vacutainer ref 369622) and stored in the dark at room temperature. All samples were analysed within 24 h. The red cells were lysed using 0.84% NH_4_Cl for 10 min at room temperature. The samples were spun in a centrifuge for 10 minutes in 1200 rpm (250 x g) and washed in PBS (without Mg^2+^ and Ca^2+^). 100 microliters of FACS buffer (PBS + 0.5% BSA) were added to the pellet. 50 microliters was transferred to each of the two FACS tubes. 50 microliters of antibody mix was added to their respective FACS tube (for antibody mixes, see below). The analyses were performed on FACSCanto II (BD Biosciences) using FACSDiva software. Cell populations were identified according to the gating strategies described by Maecker et al. [25] (Supplemental Figure 1). The T helper subsets were defined as the percentage of CD183^+^CD196^−^CD3^+^ CD4^+^ (Th1), CD183^−^CD196^−^CD3^+^ CD4^+^(Th2), or CD183^−^CD196^+^CD3^+^CD4^+^ (Th17) of the total number of CD4^+^CD3^+^ T cells. At least 50,000 lymphocytes were collected based on forward and side scatter properties. Tregs were defined as the percentage of CD127^−^CD25^+^CD45R0^+^CD194^+^ cells of the total number CD45^+^CD3^+^CD4^+^ T cells (Supplemental Figure 2).

### 2.3. Antibody Reagents

The following antibodies were used:

Antibody mix 1: CD3 PerCP Cy5.5 (BD552852), CD4 V450 (BD560345), CD8 V500 (BD560774), CD197 Alexa Flour 647 (BD557734), CD45RA PE Cy7 (BD560675), CD183 Alexa 488 (BioLegend 353710), and CD196 PE (BioLegend 353410)
Antibody mix 2: CD4 FITC (BioLegend 357406), CD25 PE (BD555432), CD194 BV421 (BioLegend 395414), CD127 Alexa Flour 647 (BD558588), CD45 V500 (BD560779), CD45 RO PeCy7 (BD337168), CD3 PerCP Cy5.5, and HLA-DR APC H7/Cy7 (BD561358)

### 2.4. Cytokines and Chemokines: Enzyme-Linked Immunosorbent Assay (ELISA)

From the 33 patients that were first included in this study and 13 HBD, EDTA-plasma was obtained and stored at −80°C until analysed. Plasma concentrations of IFN-*γ*, CXCL9, IL-4, CCL22, IL-23, and CCL20 were measured using Quantikine® ELISA from R&D Systems according to the manufactory's instructions. Plasma concentrations of IL-17A were measured using Sandwich ELISA from Affymetrix eBioscience. Optical densities were measured at 540 nm and 450 nm for IFN-*γ*, CXCL9, IL-4, CCL22, IL-23, and CCL20 and at 450 nm for IL-17A. Concentrations were calculated from standard curves created in Excel (Microsoft Office).

### 2.5. Statistical Analysis

All statistical analyses were performed on GraphPad Prism 7.0 software (GraphPad Software, San Diego, CA, USA). Spearman's correlation coefficient by rank was used to evaluate correlations between two sets of nonparametric data. Categorical variables are presented as frequencies, and continuous data are expressed as median with interquartile ranges (IQRs). For comparison of categorical data in two independent groups, Fisher's exact test or chi^2^ test was performed. For comparison between two independent groups of nonparametric data, Mann-Whitney's *U* test was performed and medians with IQRs are presented. For comparison between more than 2 independent groups with nonparametric data, Kruskal-Wallis test was performed and results are presented with medians and IQRs. *p* values below 0.05 were considered significant.

## 3. Results

### 3.1. Increased Frequency of Effector Memory T Cells in AAV

**Figure 1:**
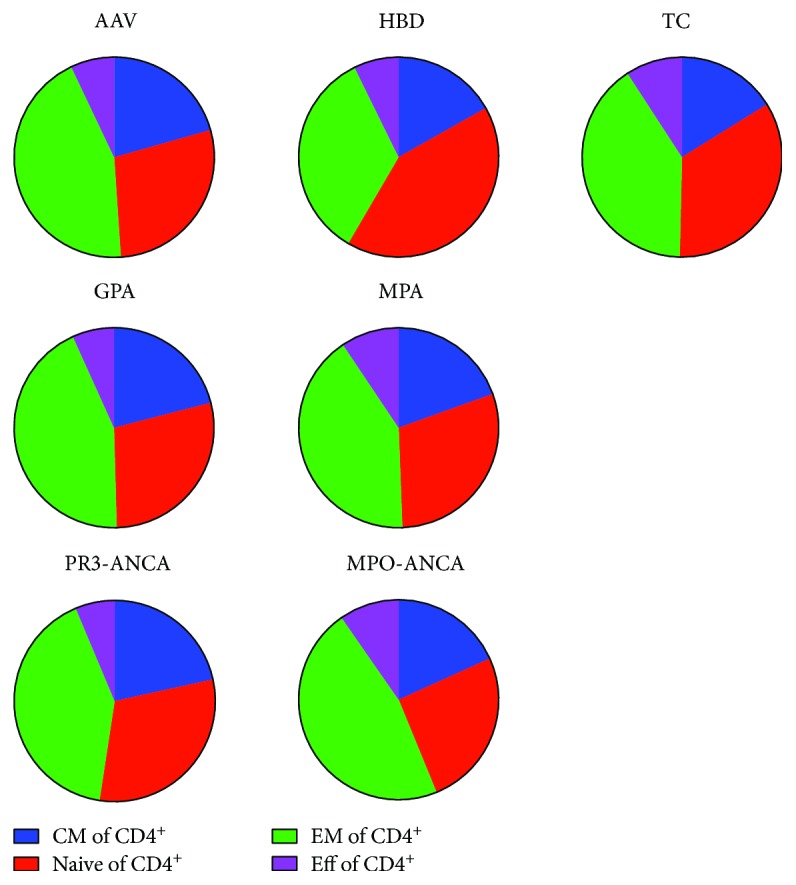
Median percentage of central memory (CM), naïve, effector memory (EM), and effector (Eff) T cells of the CD4^+^ T cell population. AAV patients have significantly lower percentage of naïve CD4^+^ T cells (*p* = 0.0016) and higher percentage of EM (*p* = 0.027) compared to HBD. No differences were detected to TC. The AAV group was divided according to clinical diagnosis (GPA and MPA) as well as ANCA specificity (PR3-ANCA and MPO-ANCA) and reanalysed. Both GPA and MPA had lower percentage of naïve CD4^+^ T cells compared to HBD (*p* = 0.0092 and *p* = 0.0425, respectively), but no significant differences for the other subpopulations were found. No differences were found between the PR3-ANCA group and MPO-ANCA, HBD, or TC. The MPO-ANCA group had a significantly smaller proportion of naïve CD4^+^ T cells (*p* = 0.0014) and a larger percentage of EM CD4^+^ T cells (*p* = 0.0107) compared to HBD.

To characterize CD4^+^ T cells in AAV patients, the distribution of naïve (CD197^+^CD45RA^+^), CM (CD197^+^CD45RA^−^), Eff (CD197^−^CD45RA^+^), and EM (CD197^−^CD45RA^−^) CD4^+^ T cells were analysed using flow cytometry. All values are given as percentage of CD4^+^ T cells. We found that AAV patients had lower percentages of naïve CD4^+^ T cells (median 26.5 IQR: 13.2, 36.7) than HBD (median 40.0 IQR: 31.6, 51.3, *p* = 0.0016) (Figure 1). In addition, a corresponding increase in the proportion of EM CD4^+^ T cells was detected in AAV patients (median 41.2 IQR: 31.5, 53.1) compared with HBD (median 33.0 IQR: 24.3, 42.7) (*p* = 0.027). This was true both when the patients were divided according to their clinical phenotype as well as by autoantibody specificity. There were no significant differences in the percentages of CM or Eff cells. TC had a similar distribution of naïve, CM, EM, and Eff cells as AAV patients.

### 3.2. Increased Percentages of Th17 Cells with a Memory Phenotype in AAV

**Figure 2:**
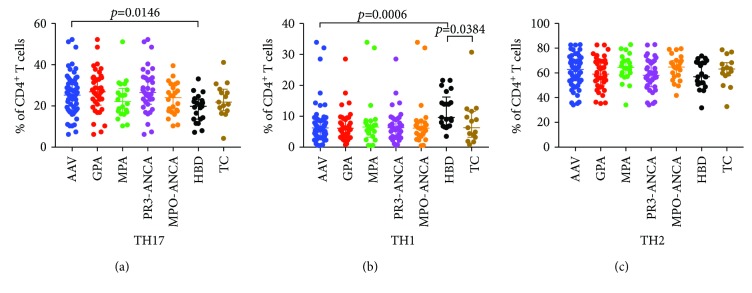
Percentages of total number of CD4^+^ T cells that are characterized as (a) Th17 cells (CD183^−^CD196^+^), (b) Th1 cells (CD183^+^CD196^−^), and (c) Th2 cells (CD183^−^CD196^−^). ANCA-associated vasculitis (AAV) and the subgroups GPA and MPA or PR3-ANCA and MPO-ANCA are compared to healthy blood donors (HBD) and therapy controls (TC). Three statistical analyses were done using the Kruskal-Wallis test followed by Dunn's multiple comparison test: first, AAV was compared to TC and HBD; second, GPA and MPA were compared to TC and HBD; third PR3-ANCA and MPO-ANCA were compared to TC and HBD. The statistically significant differences from the first analysis are indicated in the figure. When comparing GPA and MPA against HBD and TC, we found that the GPA patients had a higher percentage of Th17 compared to HBD (*p* = 0.0055) and that both GPA (*p* = 0.0031) and MPA (*p* = 0.0125) had lower percentage of Th1 cells compared with HBD. The analysis of PR3- and MPO-ANCA patients showed similar results; i.e., PR3-ANCA had significantly higher percentage of Th17 cells compared with HBD (*p* = 0.0053) and both PR3-ANCA (*p* = 0.0051) and MPO-ANCA (*p* = 0.0085) had lower percentages of Th1 cells compared with HBD.

The CD4^+^ T cells were divided in Th1 (CD183^+^CD196^−^), Th2 (CD183^−^CD196^−^), and Th17 (CD183^−^CD196^+^). Patients with AAV had higher percentages of Th17 cells (median 24.6 IQR: 17.7, 23.2) than HBD (median 19.1 IQR: 13.6, 23.2) (*p* = 0.015) (Figure 2(a)). There was no significant difference between patients with AAV compared with TC (median 23.4; IQR: 18.6, 29.5) (*p* ≥ 0.999). Both AAV and TC had lower percentages of Th1 cells compared with HBD, but there was no difference between AAV and TC (Figure 2(b)). No significant differences were observed for Th2 cells between any of the groups (Figure 2(c)).

When the patients were divided by clinical phenotype or autoantibody specificity, we found that patients with GPA had higher percentages of Th17 cells (median 27.6 IQR: 19.7, 33.0) compared with HBD (median 19.1 IQR: 13.6, 23.3, *p* = 0.0055) but not compared with TC (median 23.4 IQR: 18.6, 29.5, *p* = 0.8428). The percentages of Th17 did not differ between patients with MPA, HBD (*p* = 0.350), or TC (*p* ≥ 0.999) (Figure 2(a)). Both GPA and MPA as well as PR3-ANCA and MPO-ANCA subgroups had lower percentages of Th1 cells compared with HBD, but there was no difference between them and TC (Figure 2(b)). No differences regarding percentages of Th2 cells were observed between any of the subgroups (Figures 2(b) and 2(c)).

The Th1, Th2, and Th17 cells were further subdivided into naïve, CM, EM, and Eff cells. AAV patients had higher percentages of Th17 CM cells (*p* = 0.039) as well as higher percentages of EM Th17 (*p* = 0.020) when comparing with HBD. In addition, AAV patients had lower percentages of naïve Th1 cells (*p* = 0.0003), CM Th1 cells (*p* = 0.0026), and EM Th1 cells (*p* = 0.027) compared with HBD. Moreover, AAV patients had a decreased frequency of naïve Th2 cells (*p* = 0.0146) and increased percentages of EM Th2 cells (*p* = 0.0001) compared with HBD. No significant differences were found when AAV patients were compared with TC. For results after dividing the AAV patients in groups by disease activity and clinical phenotype, see Supplementary Table 1.

### 3.3. Disease Activity and Distribution of CD4^+^ T Cell Subsets

To investigate if disease activity affected the distribution of CD4^+^ T cell subsets, patients were divided into two groups: active disease (BVAS ≥ 1) or remission (BVAS = 0). Patients with active disease had a decreased percentage of Th1 cells (median 6.5 IQR: 3.9, 8.9) compared with HBD (median 9.7 IQR: 7.7, 18.3, *p* = 0.0072) (data not shown). However, there was no significant difference compared with TC (median 7.0 IQR: 3.2, 12.5, *p* ≥ 0.999). No differences in percentages of Th1 cells between AAV patients in remission, HBD, and TC were observed.

AAV patients in remission had significantly higher percentages of Th17 cells (median 24.9 IQR: 17.6, 31.2) than HBD (median 19.1 IQR: 13.6, 23.2, *p* = 0.0259), while there was no significant difference when comparing with TC (median 23.4 IQR: 18.6, 29.5, *p* ≥ 0.999). No differences in percentages of Th17 cells between active AAV patients (median 23.5 IQR: 18.2, 31.48), HBD (*p* = 0.095), or TC (*p* > 0.999) were observed.

Percentages of Th2 cells did not differ between active AAV (median 63.0 IQR: 53.9, 71.5), HBD (median 57.9 IQR: 46.8, 68.0, *p* = 0.602), or TC (median 61.6 IQR: 55.2, 66.4, *p* ≥ 0.999). Neither was there any significant difference between AAV patients in remission (median 56.7 IQR: 45.7, 65.6) compared to HBD (*p* ≥ 0.999) nor AAV patients in remission compared to TC (*p* = 0.589).

In summary, a decreased frequency of Th1 cells was observed in AAV patients with active disease compared to HBD, whereas AAV patients in remission had an increased portion of Th17. No significant differences between AAV patients and TC were found.

### 3.4. Percentages of Th17 Cells in Relation to Clinical Data

Since the percentage of Th17 cells was increased in AAV patients and particularly in patients with GPA and/or PR3-ANCA compared with HBD, we investigated other potential associations to clinical phenotype of the disease, renal function, and treatment with corticosteroids.

**Figure 3:**
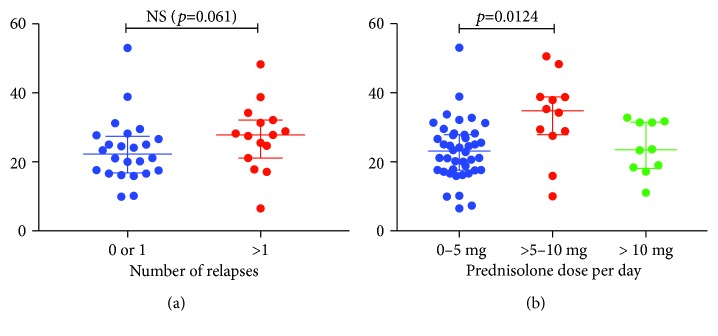
Percentage of Th17 cells (CD183^−^CD196^+^) of the total number of CD4^+^ T cells in patients with ANCA-associated vasculitis. In (a), the patients are divided according to their tendency to flare. The patients with tendency to flare also have a tendency to have higher percentage of Th17 cells compared to the patients with one or no flares. In (b), the AAV patients are divided by their prednisolone dose at the time of sampling. Patients with 5–10 mg dose per day have significantly higher levels of Th17 cells compared to patients with 0–5 mg prednisolone per day.

When comparing percentages of Th17 cells between patients with localized disease (median 27.7 IQR: 20.6, 8.2) and systemic disease (median 24.1 IQR: 17.4, 31.4), no significant difference was found (*p* = 0.447). Furthermore, to study if percentages of Th17 cells correlated with tendency to relapse, patients with a follow-up time more than one year were divided into two groups. The first group had zero or one relapse since the onset of the disease. The second group had two or more relapses since onset of the disease. The group with less than two relapses had a median percentage of Th17 cells of 22.3 (IQR: 16.8, 27.4), and the more relapse-prone group had a median percentage of Th17 cells of 27.8 (IQR: 21.1, 32.1). This difference did, however, not reach statistical significance (*p* = 0.061) (Figure 3(a)). The median follow-up time in the less relapse-prone group was 49.3 months (IQR: 28.0, 107.2) which was significantly lower than in the relapse-prone group (median: 160.4, IQR: 76.2, 189.4) (*p* < 0.0001). Percentages of Th17 cells correlated neither to eGFR (*r* = −0.083, *p* = 0.519) nor to age (*r* = −0.070, *p* = 0.575).

When dividing the AAV patients in groups according to their daily oral prednisolone dosage, percentages of Th17 cells were significantly higher in the group with 5–10 mg per day (median 34.8 IQR: 27.9, 38.8), compared to the group with 0–5 mg per day (median 23.1 IQR: 17.6, 27.9, *p* = 0.012), but in the group with more than 10 mg prednisolone per day, it was not (median 23.5 IQR: 18.1, 27.9, *p* = 0.218) (Figure 3(b)). There was no correlation between percentages of Th17 cells and daily prednisolone dosage (*r* = 0.1624, *p* = 0.204).

### 3.5. Tregs in AAV Patients, HBD, and TC

**Figure 4:**
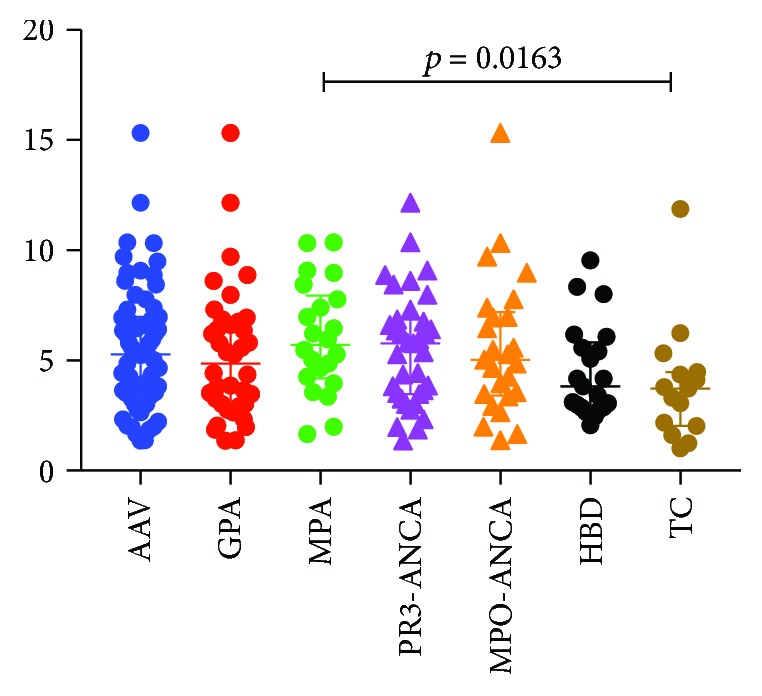
Percentage of Tregs (CD45^+^CD3^+^CD4^+^CD127^−^CD25^+^CD45R0^+^CD194^+^) of the CD4^+^ T cell population in peripheral blood from patients with ANCA-associated vasculitis (AAV), divided into GPA and MPA or PR3-ANCA and MPO-ANCA, healthy blood donors (HBD), and therapy controls (TC). Three statistical analyses were done using the Kruskal-Wallis test followed by Dunn's multiple comparison test: first, AAV was compared to TC and HBD; second, GPA and MPA were compared to TC and HBD; third, PR3-ANCA and MPO-ANCA were compared to TC and HBD. The only difference found was between MPA patients and TC.

The frequency of Tregs (CD45^+^CD3^+^CD4^+^CD127^−^CD25^+^CD45R0^+^CD194^+^) in peripheral blood was analysed by flow cytometry. No differences in the percentages of Tregs were observed between AAV patients, HBD (*p* = 0.432), or TC (*p* = 0.064) (Figure 4). However, patients with MPA had significantly higher percentages of Tregs with a median of 5.7 (IQR: 4.2, 8.0) compared with TC (*p* = 0.0163) but not when comparing to HBD (*p* = 0.113). These differences in percentages of Tregs were not seen when comparing GPA patients (median 4.9 IQR: 3.0, 6.8) with HBD (*p* ≥ 0.999) or TC (*p* = 0.246). The percentages of Tregs did not show any difference when the AAV patients were divided according to ANCA specificity (Figure 4). We did not find any differences associated with disease activity (data not shown).

### 3.6. Elevated Concentrations of CXCL9 and CCL20 in AAV Patients

Cytokine and chemokine profiles were analysed in plasma to see if we could find any association with a Th1, Th2, or Th17 profile. Seven selected cytokines and chemokines were analysed in EDTA plasma, collected at the same time as the CD4^+^ T cell subsets were evaluated by flow cytometry, from the first included 33 AAV patients and 13 HBD.

The concentrations of the Th1-related chemokine CXCL9 were increased in plasma from AAV patients compared with HBD (Table 3). This difference remained when dividing the AAV group in active and inactive disease, as well as when dividing in GPA and MPA. In addition, the plasma concentrations of the Th1-associated cytokine IFN-*γ* were higher in MPA patients compared with HBD. Furthermore, plasma concentrations of the Th17-related chemokine CCL20 were elevated in AAV patients compared with HBD (Table 3). Notably, there was a correlation between concentration of CCL20 and absolute counts of Th17 cells calculated from the white blood cell counts (*p* = 0.0125, *r* ^2^ = 0.22, linear regression analysis). No differences were found in plasma concentrations of IL-4, CCL22, IL-23, or IL-17A in patients with AAV compared to HBD.

### 3.7. Decreased T Cell Count in AAV Patients

Absolute T cell counts were calculated from routine white blood cell counts multiplied with the number of events in the CD3^+^ gate divided by the number of events in the CD45^+^ gate. As no blood cell counts were performed on the HBD, all comparisons are done against the reference intervals (based on 50 healthy blood donors and presented as the 2.5 to the 97.5 percentile) from the clinical immunology lab at Skåne University Hospital. Both AAV and TC had low CD3^+^ T cell counts (AAV median 0.35 IQR 0.2, 0.76, TC median 0.72 IQR 0.31, 1.26 ×10^9^ cells/L) compared to the reference values (0.6–2.1 × 10^9^ CD3^+^ cells/L 2.5–97.5 percentile). There was a tendency that the AAV patients had lower counts than TC, but it did not reach statistical significance. The frequency of both AAV and TC that have CD3^+^, CD4^+^, and CD8^+^ T cell counts below the lower reference value (2.5^th^ percentile) from the clinical immunology lab is significantly higher than expected (*p* < 0.0001, chi-square test) (Supplementary Figure 3). Nonetheless, all samples had T cell counts high enough to give reliable results also when analysing the smaller T cell subpopulations.

## 4. Discussion

The aim of this project was to phenotypically characterize CD4^+^ T cells in AAV patients and to investigate if this correlate with clinical data, e.g., clinical diagnosis, ANCA specificity, age, eGFR, disease activity, and therapy. In addition, to indirectly estimate their function and activity, circulating plasma concentrations of some cytokines and chemokines associated with certain CD4^+^ T cells subsets were analysed. Earlier studies have suggested an increased and persistent T cell activation in AAV; however, these studies have mainly focused on GPA [26, 27].

### 4.1. Th17 Cells, IL-17A, and CCL20 in AAV

In line with earlier studies [17, 28], we found an increased frequency of circulating Th17 cells in patients with GPA compared to HBD. When dividing the patients by autoantibody specificity, patients with PR3-ANCA had higher percentages of Th17 cells than HBD. Th17 cells and their related cytokines have not been well studied in MPA. Gan et al. found evidence that Th17 cells are crucially involved in development of glomerulonephritis in MPO-immunized mice [20]. In our study, we could not show elevated percentages of Th17 cells in patients with MPA. As all our measurements are done on circulating cells, involvement of Th17 cells in the local inflammatory responses in MPA cannot be ruled out and further studies at inflammatory sites are needed.

In this study, plasma concentrations of IL-17A were similar in AAV patients compared to HBD. In contrast, the plasma concentrations of the Th17-related chemokine CCL20 were found to be elevated. Interestingly, concentrations of CCL20 were higher in both GPA and MPA patients compared to HBD. This is in line with the findings presented by Eriksson et al. who also found elevated levels of circulating CCL20 in patients with GPA compared with HBD and, in similarity to our results, did not find any significant difference regarding levels of IL-17A [29]. In another study by Nogueira et al., levels of IL-17A were found to be elevated in about one third of the patients compared with HBD [18].

We could not show that the percentage of Th17 cells varies with disease activity estimated by BVAS, and it was not significantly different between patients with localized disease compared to systemic disease. There was, however, a tendency to higher levels in the group with a relapse-prone disease. Wilde et al. did not find any difference when comparing relapsing GPA patients to relapse-free patients [28]. There are several differences between the studies that can explain the somewhat deviating results: in Wilde et al.'s study, all samples came from untreated patients and the definitions of the two groups were different. A limitation in our study is the significant difference regarding follow-up time between the more relapse-prone group and the group with zero or one relapse during the follow-up time.

Regarding corticosteroid treatment, patients with a daily dosage of >5–10 mg prednisolone had higher percentages of Th17 cells than the patients with 0–5 mg a day as well as the patients with more than 10 mg a day. The group of patients with >5–10 mg prednisolone a day consisted of GPA, MPA, and EGPA patients either in remission or with grumbling disease. Patients with more than 10 mg prednisolone per day were mainly patients with active disease (Table 1). Wilde et al. found a negative correlation between circulating levels of Th17 and steroid dosage in both active and quiescent GPA [28]. Our group of patients was very heterogenic regarding treatment with other immunosuppressants, and some of the patients with active disease had received cyclophosphamide which may have affected the results. The potential effects of cyclophosphamide on Th17 cells in AAV are, however, not well established.

### 4.2. Th1 and Th2 Cells and Their Main Cytokines in AAV

We did not find any significant differences in percentages of Th1 cells between PR3- and MPO-ANCA patients, or between MPA and GPA patients. Moreover, there was no difference in Th1 cells in patients in remission compared to HBD and TC. However, patients with active disease showed lower percentages of Th1 cells than HBD, but not compared to TC. No differences in levels of Th1 cells were found between AAV patients and HBD in a study by Abdulahad et al., but they had only included patients in remission [17]. In line with results from Nogueira et al., circulating concentrations of the Th1-associated cytokine IFN-*γ* did not differ between patients with active AAV, quiescent AAV, or GPA compared to HBD [18]. In our study, patients with MPA had slightly higher concentrations of circulating IFN-*γ* than HBD. Interestingly, significantly higher concentrations of the Th1-related chemokine CXCL9 were found in AAV, both GPA and MPA—regardless of disease activity, compared to HBD.

Percentages of Th2 cells did not differ between AAV patients either with active disease or in remission compared to HBD and TC. Neither was there a difference between patients with GPA or MPA compared to HBD and TC. This stands in contrast to findings by Abdulahad et al., who showed an expansion of IL-4^+^ T cells in patients with GPA in remission [17]. In our study, circulating concentrations of the regulatory Th2 cytokine IL-4 and Th2-associated chemokine CCL22 did not differ between patients with active disease, quiescent disease, and HBD, and there were no significant differences between patients with GPA or MPA, respectively, compared to HBD. Eriksson et al. found decreased levels of circulating CCL22 in patients with active AAV compared with patients in remission as well as HBD [29]. As mentioned above, our group of patients with active disease was small and heterogeneous, which may explain the differences in the results.

### 4.3. Memory T Cells Are Expanded in AAV

When dividing the CD4^+^ T cells into subsets based on their activation and differentiation, AAV patients had significantly lower percentages of naïve CD4^+^ T cells and higher percentages of effector memory CD4^+^ T cells compared to HBD. Similar results were found by Abdulahad et al. regarding GPA patients in remission, and they suggested that in AAV, a strong and continuous stimulation of naïve CD4^+^ T cells entails a differentiation towards EMT cells [21]. In turn, this could contribute to the relapsing course of the disease, which is also suggested in a review by Wilde et al. in 2010 [30]. In our study, CM Th17 as well as EM Th17 were expanded in patients with AAV compared with HBD. This was true for the AAV group as a whole but also for patients in remission compared to HBD, supporting the idea of an expansion of memory cells due to persistent proinflammatory stimuli.

### 4.4. Tregs in AAV

Functionally defect Tregs have been suggested to play a role in the pathogenesis of AAV, since they control and diminish inflammatory responses by other T cells [22, 31]. In a recent study, Szczeklik et al. found a decrease in Tregs in patients with active GPA compared to HBD [32]. Abdulahad et al. found an expansion of Tregs in patients with quiescent GPA, but these cells showed a reduced or absent suppressive function [22]. In our study, we did not find any differences regarding percentage of circulating Tregs in AAV as compared to HBD or TC. We have chosen to use standardized antibody panels as suggested by the Human Immunophenotyping Consortium [25], and this panel does not include FoxP3 as most previous studies have included making direct comparisons hard. Moreover, in our study, we have only measured circulating percentages of Tregs and not the function or activity of these cells. Studies have shown that Tregs in the presence of proinflammatory cytokines can differentiate into IL17-producing cells, why a reciprocal relationship between Th17 cells and Tregs has been suggested [33, 34], but the present study is not designed to be able to illustrate this. The diversity of and phenotypic overlap among various T cell subsets including Tregs was recently shown by Kunicki et al. [35] using single-cell mass cytometry; hence, the use of different flow cytometry strategies might explain differences between in general, including ours.

### 4.5. Altered CD4^+^ T Cell Distribution in AAV: A Result from Treatment or the Disease?

It is of importance that even though some CD4^+^ T cell phenotypes have been expanded in AAV patients in comparison to HBD, they do not significantly differ from therapy controls. In parallel to the group of patients with active AAV, the TC group is also somewhat clinically heterogenic which can influence the results. Furthermore, CD4^+^ T cells are important in antibody-mediated rejection of kidney transplants [36, 37] and all of the TC were treated with calcineurin inhibitors which have earlier been found to affect the levels of CD4^+^ T cells [38, 39]. One could hypothesize that this, as in AAV, results in an altered distribution of CD4^+^ T cells, depending on the balance between inflammatory responses due to the transplantation and immunosuppressive therapies. To our knowledge, earlier studies on involvement of CD4^+^ T cells in AAV have not included therapy controls. We believe that it is of great importance to include therapy controls in order to be able to determine whether the differences are a result of the studied disease or related to treatment and/or other factors.

## 5. Conclusions

In this study, we found a skewed balance of CD4^+^ T cells in AAV patients, confirming previous results from studies of GPA patients. AAV patients have elevated percentages of Th17 cells, lower percentages of Th1 cells, larger proportion of EM, and fewer naïve T cells compared to healthy controls. This seems to result in, for instance, higher plasma concentrations of the proinflammatory chemokine CCL20.

Interestingly, almost no differences were found when comparing the AAV group to the TC group. This leads to the question: is the skewed balance of CD4^+^ T cells in AAV patients a result of therapy rather than disease-specific mechanisms? To finally answer this question, new studies are needed including larger number of AAV patients and TC that are both age- and sex-matched.

## Figures and Tables

**Table 1:** Descriptive statistics of GPA and MPA (numbers or median with lower and upper quartiles) at time of blood sampling.

	GPA	MPA	*p* value
Age in years	66.8 (50.9, 76.5)	75.7 (68.8, 82.4)	*p* = 0.0024^1^
Sex (numbers)			
Female	18	11	*p* ≥ 0.999^2^
Male	20	11	
Age at diagnosis in years	57.5 (35.9;67.3)	64.2 (73.2;76.2)	*p* ≤ 0.0001^1^
Follow-up time in months	81.9 (28.2;157.4)	16.7 (1.2;74.8)	*p* = 0.002^1^
ANCA specificity (numbers)			
PR3	30	5	*p* ≤ 0.0001^2^
MPO	7	17	
Disease activity (numbers)			
Active disease	8	10	*p* = 0.0781^2^
Remission	30	12	
Time since onset of the last flare in months	34.4 (11.4;63.2)	8.9 (0.9;34.3)	*p* = 0.060^1^
Disease phenotype (numbers)			
Localized disease	7	6	*p* = 0.520^2^
Systemic disease	31	16	
Organ involvement (numbers)			
Ear-nose-throat	30	1	*p* ≤ 0.0001^2^
Pulmonal	23	9	*p* = 0.18^2^
Renal	28	21	*p* = 0.04^2^
Central nervous system	4	1	*p* = 0.643^2^
Peripheral nervous system	4	5	*p* = 0.267^2^
Gastrointestinal	2	1	*p* ≥ 0.999^2^
Cardiovascular	1	3	*p* = 0.135^2^
BVAS (Birmingham Vasculitis Activity Score) (numbers)			*p* = 0.05^3^
0	30	12	
1–5	3	1	
6–10	4	4	
>10	1	5	
Vasculitis Damage Index	2 (1, 4)	2 (0, 3)	*p* = 0.291^1^
CRP (mg/L)	3.4 (1.4, 6.1)	2.4 (1.4, 9.5)	*p* = 0.954^1^
Haemoglobin (g/L)	129.0 (122.5, 138.0)	124.0 (111.0, 133.5)	*p* = 0.231^1^
Platelets (10^9^/L)	263.0 (204.3, 313.3)	230.0 (183.5, 287.5)	*p* = 0.159^1^
White blood count (10^9^/L)	6.6 (5.2, 8.6)	7.3 (5.4, 8.4)	*p* = 0.881^1^
Creatinine (micromoles/L)	101.0 (72.5, 141.0)	158.0 (106.0, 199.3)	*p* = 0.002^1^
eGFR (mL/min/1.73 m^2^)	64 (45, 86)	33 (21, 48)	*p* = 0.0001^1^
Prednisolone (mg/day)			*p* = 0.006^3^
0	11	7	
2.5–5	16	5	
>5–10	10	2	
>10–20	1	4	
>20	0	4	
Azathioprine	12	8	*p* ≥ 0.999^2^
Median dose (mg/day)	125 (range: 50, 200)	75 (range: 50, 100)	*p* = 0.100^1^
Mycophenolate mofetil	5	0	*p* = 0.148^2^
Median dose (mg/day)	2000 (range: 250, 2500)	NA	
Methotrexate	5	0	*p* = 0.148^2^
Median dose (mg/week)	25 (range: 15, 25)	NA	
Cyclophosphamide po	0	0	*p* = 0.528^2^
Cyclophosphamide iv (within 6 months)	4	7	*p* = 0.029^2^
Median time since last infusion (days)	83.5 (31.8, 103.0)	18 (12.3, 135.8)	*p* = 0.504^1^
Rituximab (ever)	14	3	*p* = 0.077^2^
Median time since last infusion (days)	367.0 (139.5, 902.5)	316.0 (91.0, 973.0)	*p* = 0.953^1^

GPA = granulomatosis with polyangitis; MPA = microscopic polyangitis. ^1^Mann-Whitney test, ^2^Fisher's exact test, and ^3^chi-square test.

**Table 2:** Descriptive statistics of AAV, HBD, and TC (numbers or median with lower and upper quartile) at time of blood sampling.

	AAV^∗^	HBD	TC^∗^	*p* value
Age in years	70.21 (53.84, 73.39)	56.0 (IQR: 45.3, 60.2)	49.41 (40.99, 63.03)	*p* = 0.021^1^
Sex (numbers)				
Women	31	11	4	*p* = 0.252^2^
Men	33	10	11	
Prednisolone median dose (mg/day)	5.0 (range: 2.5, 70.0)	NA	5.0 (range: 2.5, 5.0)	*p* = 0.0024^3^
Azathioprine median dose (mg/day)	100 (range: 50.0, 200)	NA	50 (range: 50.0, 50.0)	*p* = 0.25^3^
Mycophenolate mofetil median dose (mg/day)	2000 (range: 250, 2500)	NA	1500 (range: 750, 2000)	*p* = 0.52^3^

AAV = anti-neutrophil cytoplasmic autoantibody- (ANCA-) associated vasculitides; TC = therapy control. ^1^Kruskal-Wallis test by ranks, ^2^chi-square test, and ^3^Mann-Whitney test. ^∗^In this table, only the drugs that AAV and TC have in common are disclosed.

**Table 3:** Concentrations of CD4^+^ T cell-related cytokines and chemokines in plasma from patients with ANCA-associated vasculitis (AAV) and healthy blood donors (HBD).

	IFN-*γ*	CXCL9	IL-4	CCL22	IL-23	IL-17A	CCL20
AAV *n* = 33	13.6^∗^ (12.8, 14.6)	61.5^∗∗^ (38.9, 79.7)	6.21 (6.21, 7.19)	248.1 (171.2, 398.8)	53.8 (50.3, 57.3)	7.51 (7.10, 8.44)	28.6^∗∗^ (21.4, 39.5)
Active AAV *n* = 7	13.6 (12.8, 14.4)	60.4^∗∗^ (43.4, 65.0)	6,21 (4.43, 7.0)	221.4 (34.9, 248.1)	52.6 (50.3, 57.3)	7.92 (7.10, 8.23)	35.8^∗∗^ (28.6, 37.2)
Inactive AAV *n* = 26	13.8 (12.8, 14.8)	64.0^∗∗^ (37.0, 80.1)	6,66 (6.21, 7.32)	258.3 (174.8, 507.2)	53.5 (50.3, 57.3)	7.46 (7.07, 8.64)	21.3^∗∗^ (21.3, 0.434)
GPA *n* = 21	13.2 (12.8, 14.4)	51.8^∗∗^ (37.0, 69.1)	7.10 (6.21, 7.55)	239.9 (190.7, 359.8)	53.8 (50.9, 57.3)	7.82 (7.20, 8.64)	25.3^∗∗^ (18.5, 35.8)
MPA *n* = 10	14.0^∗^ (12.9, 16.0)	72.5^∗∗^ (56.1, 117.4)	6.21 (5.10, 6.43)	258.3 (73.3, 647.9)	53.8 (50.3, 60.9)	7,05 (7.00, 8.15)	38.2^∗∗^ (30.5, 74.1)
HBD *n* = 13	12.8 (12.2, 13.2)	22.1 (19.5, 30.4)	6.21 (6.21, 7.99)	252.2 (180.4, 307.5)	53.8 (50.3, 56.1)	7.72 (7.20, 8.18)	16.2 (15.0, 18.1)

Concentrations are measured in pg/mL. Figures represent median with interquartile ranges. ^∗^ *p* ≤ 0.05 and ^∗∗^ *p* ≤ 0.01, when compared with HBD.

## Data Availability

Flow cytometry raw data files can be obtained as FACS version 3.1 files from the corresponding author.

## Supplementary Materials

Supplementary Materials Supplementary Table 1 shows percentages of the different CD4^+^ T cell subsets in different patient subgroups. Supplementary Figures 1 and 2 show the gating strategies that were used to identify the different T cell subpopulations. Supplementary Figure 3 shows the calculated absolute numbers of T cells in blood from both patients and therapy controls.
