# Supplementary material for: Phenotypic Characterization of Circulating CD4+ T Cells in ANCA-Associated Vasculitis
Source: J Immunol Res. 2018 Oct 30;2018:6984563. doi: 10.1155/2018/6984563 (PMC6232803; doi:10.1155/2018/6984563)
Supplement: Supplementary Materials — Supplementary Table 1 shows percentages of the different CD4+ T cell subsets in different patient subgroups. Supplementary Figures 1 and 2 show the gating strategies that were used to identify the different T cell subpopulations. Supplementary Figure 3 shows the calculated absolute numbers of T cells in blood from both patients and therapy controls. [file 6984563.f1.pdf]

Supplemental table and figures

Table S1

	Active AAV	Inactive AAV	GPA	MPA	HBD	TC
**Th1 naive**	0.65 (0.35;1.00)**	0.65 (0.30;1.25)**	0.60 (0.30;1.03)**	0.75 (0.20;1.70)*	1.40 (0.95;2.70)	0.3 (0.20;0.70)
**Th1 CM**	1.50 (0.75;1.98)*	1.35 (0.68;2.38)*	1.30 (0.78;2.45)*	1.50 (0.57;1.90)**	2.50 (1.85;3.20)	1.90 (0.50;2.80)
**Th1 EM**	2.45 (1.68;3.80)	2.80 (1.88;4.50)	2.60 (1.60;3.73)*	2.65 (1.88;3.80)	4.50 (2.65;8.45)	3.50 (1.90;5.50)
**Th1 EFF**	0.40 (0.28;0.63)	0.75 (0.3;1.63)	0.60 (0.30;1.63)	0.55 (0.30;1.75)	1.00 (0.45;2.05)	0.40 (0.20;0.90)
**Th2 naive**	29.55 (21.73;35.55)	18.70 (10.1;33.93)	23.1 (11.65;39.95)	26.55 (15.35;33.85)	33.00 (27.55;46.65)	30.20 (20.20;37.90)
**Th2 CM**	9.40 (15.88;21.13)*#	7.8 (4.58;10.30)	7.95 (4.58;10.83)	8.35 (5.25;12.63)	6.60 (3.90;7.20)	5.80 (4.40;6.50)
**Th2 EM**	16.45 (10.60;21.13)	16.35 (10.64;27.25)	13.75 (9.70;23.85)**	18.60 (14.53;25.45)**	6.90 (5.85;9.25)	15.90 (8.00;23.6)
**Th2 EFF**	5.45 (2.66;9.25)	5.15 (3.18;12.88)	5.00 (2.98;12.08)	7.25 (3.83;12.28)	4.70 (2.85;7.45)	6.80 (4.00;10.70)
**Th17 naive**	0.75 (0.50;1.70)	0.80 (1.50;1.50)	0.95 (0.50;1.53)	0.80 (0.58;1.10)	1.00 (0.70;1.10)	0.40 (0.40;1.00)
**Th17 CM**	7.85 (5.73;10.78)	6.80 (4.78;9.60)	7.60 (6.28;10.78)*	7.30 (5.13;8.33)	5.40 (4.00;7.25)	5.70 (4.50;9.60)
**Th17 EM**	13.60 (9.60;18.63)	16.70 (11.18;20.93)*	17.05 (10.10;20.93)*	13.60 (10.90;17.88)	11.30 (3.75;14.00)	14.40 (11.70;20.60)
**Th17 EFF**	0.45 (0.20;0.73)	0.40 (0.20;0.73)	0.40 (0.20;0.80)	0.45 (0.20;0.68)	0.50 (0.30;0.65)	0.30 (0.20;0.50)

Table S1. Distribution of subsets of $CD4^+$ T cells (percentages of $CD4^+$ T cells) in patients with active ANCA-associated vasculitis (AAV), patients in remission, patients with Granulomatosis with polyangiitis (GPA), patients with Microscopic polyangiitis (MPA) compared to HBD and TC. Central memory (CM), Naïve, Effector memory (EM) and Effector (Eff) T cells. $^{*}p\leq0.05$, $^{**}p\leq0.01$ compared to HBD $^{\#}p\leq0.05$, $^{\#\#}p\leq0.01$ compared to TC

Figure S1

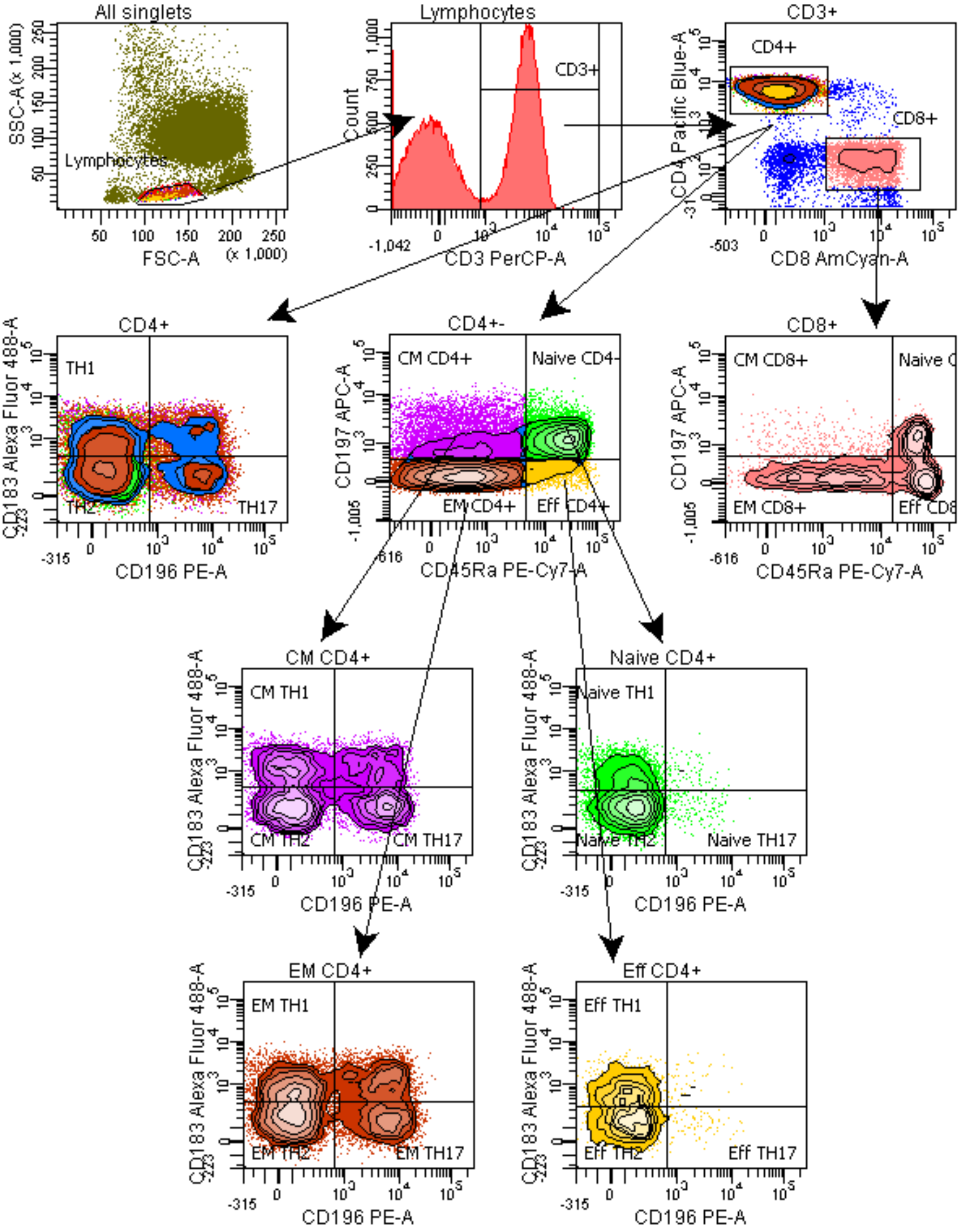

Figure S1: Gating strategies for TH1, TH2 and TH17 cells. Lymphocytes were gated from forward and side scatter plots. CD3+ lymphocytes were divided into CD4+ and CD8+ cells and further analysed based on their expression of CD197 and CD45Ra into Central memory (CM), Naïve, Effector memory (EM) and Effector (Eff) T cells. Both the entire CD4+ populations as well as CD4+ EM, Naïve, CM and Effector T cells were gated based on CD196 and CD183 into TH1, TH2 and TH17 cells. The figure shows an example from a healthy blood donor.

Figure S2

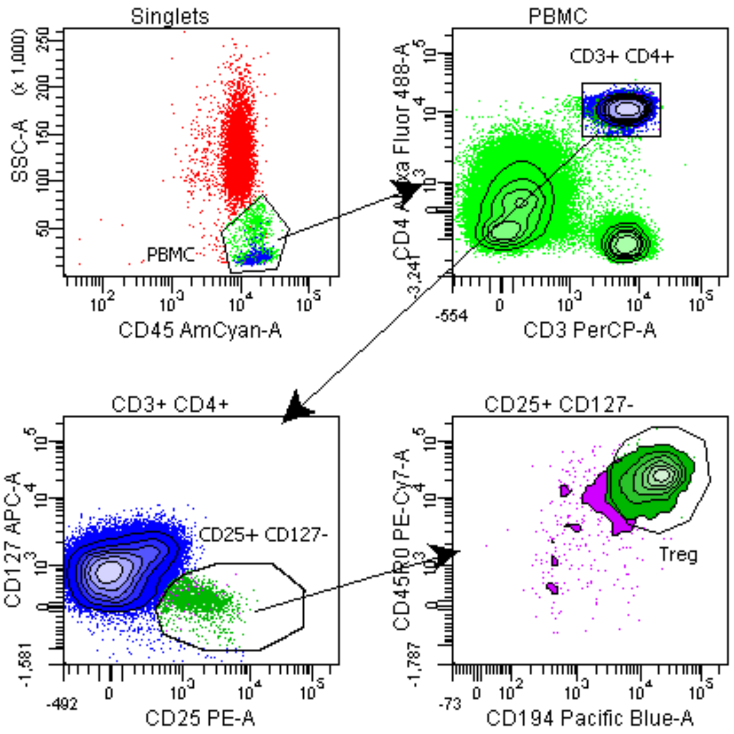

Figure S2: Gating strategies for regulatory T cells (Treg) analysis. PBMC were gated from the single cell population, as determined by forward height and area linearity. The PBMC population were further gated using CD3/CD4, CD25/CD127 and CD45RO/CD194 plots as shown above. Tregs were defined as CD3+CD4+CD25+CD127-CD194+CD45R0+ cells. The figure shows an example from a healthy blood donor.

Figure S3

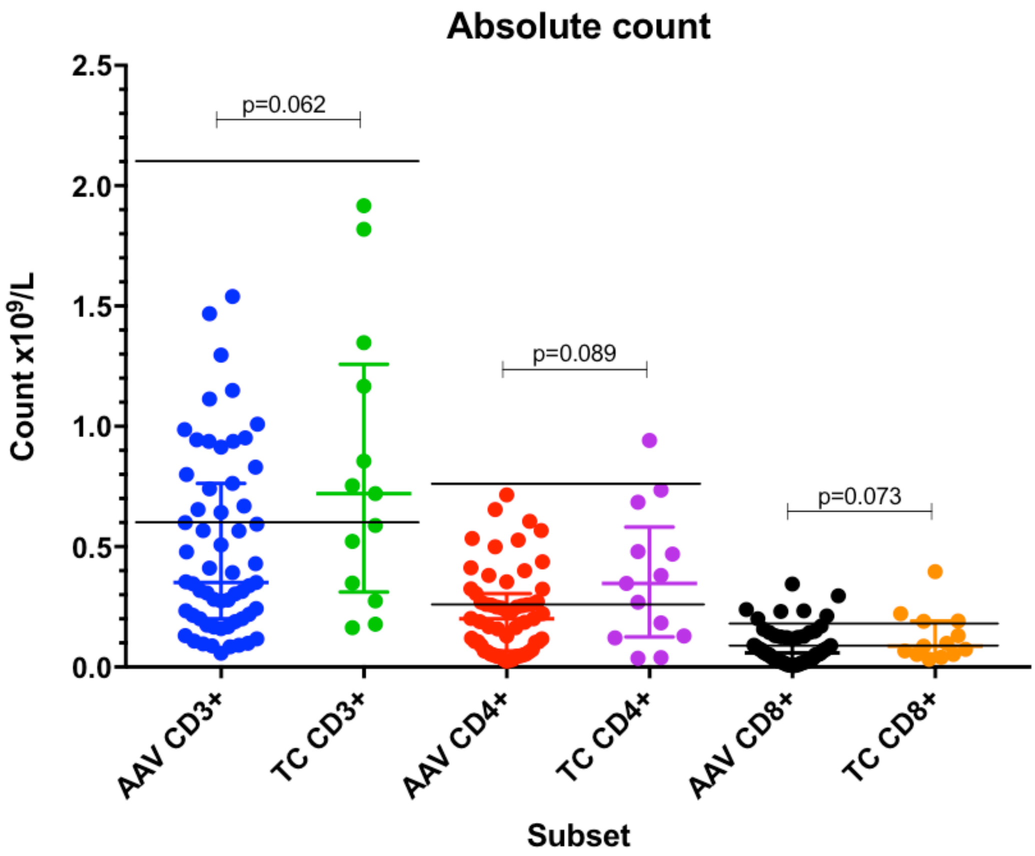

Figure S3: Absolute counts of all T cells ($CD3^+$), $CD4^+$ T cells and $CD8^+$ T cells are calculated from white blood counts. Reference interval for healthy controls are indicated with black lines. p-values for comparisons (Mann-Whitney) are indicated. No statistical differences were found between AAV and TC but there is a tendency that AAV patients have lower count of T cells. The frequency of counts below the lower reference value (2.5 percentile) are significantly higher in both AAV and TC for all T cell subpopulations (Chi-square test).
